# *Cytospora paraplurivora* sp. nov. isolated from orchards with fruit tree decline syndrome in Ontario, Canada

**DOI:** 10.1371/journal.pone.0279490

**Published:** 2023-01-11

**Authors:** Evgeny Ilyukhin, Hai D. T. Nguyen, Alan J. Castle, Walid Ellouze

**Affiliations:** 1 Agriculture and Agri-Food Canada, Vineland Station, Ontario, Canada; 2 Agriculture and Agri-Food Canada, Ottawa, Ontario, Canada; 3 Department of Biological Sciences, Brock University, St. Catharines, Canada; Friedrich Schiller University, GERMANY

## Abstract

A new species of *Cytospora* was isolated from cankered wood of *Prunus* spp. during a survey of orchards exhibiting symptoms of fruit tree decline syndrome in southern Ontario, Canada. We found isolates that are morphologically similar to species in the *Cytosporaceae* family, which is characterized by single or labyrinthine locules, filamentous conidiophores or clavate to elongate obovoid asci and allantoid, hyaline conidia. Multi-gene phylogenetic analysis of ITS, LSU, *act* and *tef1- α* showed that the isolates form a distinct clade, sister to *Cytospora plurivora*. Morphologically, our isolates showed differences in the length of conidia and culture characteristics compared to *C*. *plurivora*, suggesting the establishment of a new species. The species is described as *Cytospora paraplurivora* sp. nov. and placed in the family *Cytosporaceae* of *Diaporthales*. Additionally, we sequenced, assembled and characterized the genome of the representative isolate for this new species. The phylogenomic analysis confirms the species order and family level classification. *C*. *paraplurivora* sp. nov. has the potential to severely affect stone fruits production, causing cankers and dieback in stressed trees, and eventually leads to tree decline. Pathogenicity tests show that the species is pathogenic to *Prunus persica var*. *persica*.

## Introduction

**Fig 1:**
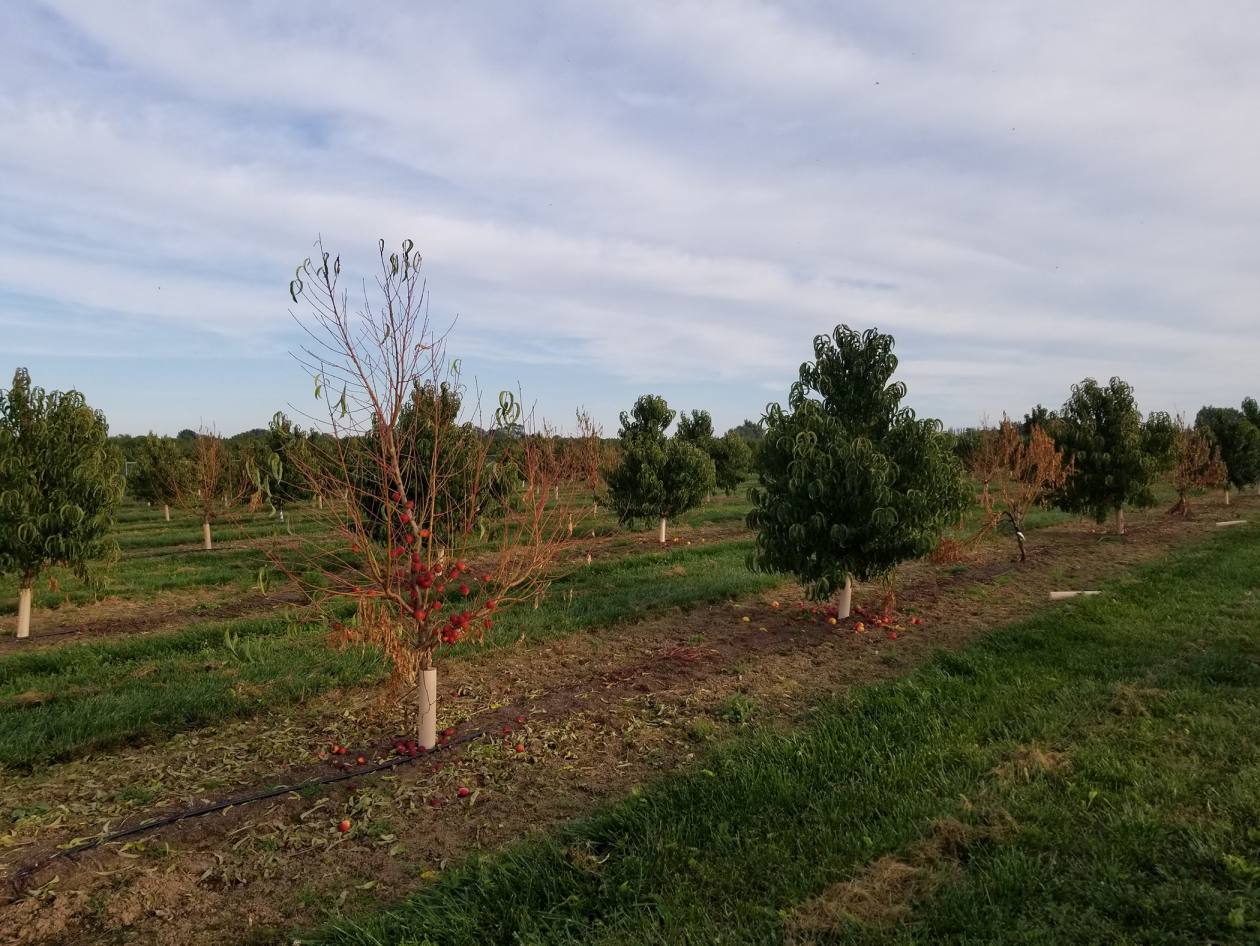
Nectarine trees showing fruit tree decline syndrome (FTDS) in southern Ontario, Canada.

Stone fruit trees are economically important crops cultivated in Ontario, Canada. The Niagara Peninsula produces more than 90% of its peaches, nectarines and apricots [1]. Peach occupy the largest production areas, followed by nectarines and apricots. Fruit tree decline syndrome (FTDS) has been recently discovered in several orchards in this area of the province. The common symptoms include stem canker and dieback, wilting, increased suckering, leaf discolouration and eventual total collapse of the tree. Disease incidence ranged from 49 to 72% for apricots and nectarines (Fig 1). Similar disease symptoms were observed in stone fruits and apple trees in other regions of North America [2, 3]. A number of studies reported that *Cytospora* spp. was among the causative agents of canker and dieback diseases in fruit trees including *Prunus* spp. [4, 5]. Generally, members of the *Cytospora* spp. infect trees that are stressed by extreme weather conditions (drought or freezing), and by invading wounds in the bark caused by insect damage and improper pruning. These fungi commonly overwinter in a form of pycnidia embedded in the bark of cankered branches [6].

The genus *Cytospora* (*Cytosporacea*, *Diaporthales*) was established by Ehrenberg in 1818. There are currently 676 records of *Cytospora* spp. registered in the IndexFungorum (www.indexfungorum.org; accessed Nov 2021). However, 163 species of the genus with available nucleotide sequences are listed in the NCBI taxonomy database (www.ncbi.nlm.nih.gov/taxonomy; accessed May 2022). Approximately, 150 species in the genus have been reported to cause diseases in more than 120 woody plants, which can result in significant commercial losses for growers [7–9]. The taxonomic species identification of *Cytospora* spp. is mainly based on morphological characteristics and molecular phylogenetic analysis. Recent studies described a number of new species of *Cytospora* on different hosts using multi-gene (ITS, LSU, *act*, *tef1- α*, *tub2*, and *rpb2*) phylogenetic analysis [5, 10–12]. This approach allows for the identification of cryptic and novel species within the genus. Obtaining high-quality, cost-effective genomic data is now possible through rapidly developing sequencing technologies. Whole-genome sequence data used in phylogenomics can contribute to resolving taxonomic uncertainties or support species reclassifications [13]. One such study found that a polyphagous plant pathogen, *Corynespora olivacea* was previously misclassified on family level [14].

The objective of this paper was to identify and characterize a novel *Cytopora* species associated with the decline of *Prunus armeniaca*, *P*. *persica* var. *persica*, *P*. *persica* var. *nucipersica* in Ontario, Canada. The characterization of the new species was performed following recently published guidelines [15, 16].

## Materials and methods

### Sample collection and isolation

Wood samples were collected in 2018–2021 from 30 apricot, 6 peach and 6 nectarine trees exhibiting extensive tree fruit decline symptoms from nine commercial orchards located in southern Ontario. Roots were symptom-free. The cankered and diseased wood sections from trunk and rootstock were cut into 1 cm pieces, and surface disinfested with 70% ethanol for 30 sec, followed by 1% NaClO for 20 min and three rinses in sterile distilled water. The samples were air-dried and placed on a 2% potato dextrose agar (PDA, Difco, USA) supplemented with kanamycin (50 mg L−1). The PDA plates were incubated at 22°C for 5 days in the dark. All fungal colony-forming units were hyphal-tip transferred to individual PDA plates and incubated at 22°C for 7 days in the dark. Purified mycelial isolates were classified into morphotypes prior to molecular identification. *C*. *paraplurivora* was characterized by fast-growing, white to cream with uneven lobate growth margin colonies.

Tree branches with fruiting structures (conidiomata) were checked separately. Single conidia isolations were performed using the protocol described by Chomnunti et al. [17]. For long-term preservation, fungal cultures were stored at −80°C in 30% glycerol. The holotype specimen was deposited in the Canadian National Mycological Herbarium (DAOM) and the living culture collection maintained by the Canadian Collection of Fungal Cultures (DAOMC).

### Morphological examination

Conidiomata formed on the tree branches were described prior to sectioning with a sterile surgical scalpel. The macro-morphological structures were measured using a dissecting microscope (OMAX 2000X Infinity Compound Siedentopf Microscope with a Built-in Camera). In order to calculate the mean size of the structures, 10 conidiomata, 20 conidiogenous cells and 50 conidia were measured. The obtained measurements were recorded with minimum and maximum values and means were calculated. Radial growth of fungal colonies was estimated with two perpendicular measurements after 5 days of incubation. Morphology description [18] and color characterisation [19] were performed after 7 days.

### DNA extraction, PCR amplification and Sanger sequencing

Genomic DNA was extracted from mycelium using the Plant/Fungi DNA Isolation Kit (Norgen Biotech Corp., Thorold, Canada) with the following modifications: fungal tissue was vortexed for 15 minutes with 1 mm glass beads and 500 μL lysis buffer and 1 μL RNase A prior to incubation at 65°C. After incubation on ice, the fungal mixture was centrifuged at 10,000 rpm to separate the lysate from the beads and biomass. During the column wash, the resin was dried by spinning for 10 minutes at 14,000 rpm. DNA was eluted at 10,000 rpm for 2 minutes. PCR amplifications were executed using C1000 Touch PCR thermal cycler (Bio-Rad, Hercules, USA) under conditions described in the references for each region. The internal transcribed spacer (ITS) region was amplified with the primer pair ITS1/ITS4 [20]. The primer pair LR0R/LR5 [21] was used to amplify the large subunit rRNA gene (LSU). The partial actin (ACT) region was amplified with the primer pair ACT512F/ACT783R [22], and the primer pair EF1-728F/ EF1-986R [22] was used to amplify partial translation elongation factor 1-alpha (*tef1-α*) gene sequences. The quality of the PCR products was examined using electrophoresis in 1% agarose gel. Sanger sequencing was carried out at Genome Quebec’s Sequencing Facility (Montreal, Canada).

### Sequence alignment and phylogenetic analysis

The consensus sequences were built from Sanger chromatograms using BioEdit [23]. The ITS, LSU, *act*, *tef1-α* sequences from our study were uploaded on GenBank (http://www.ncbi.nlm.nih.gov/) on the Basic Local Alignment Search Tool (BLAST) [24] to find other similar sequences. The sequences of *Cytospora* spp. from the top BLAST hits were then extracted and added to recently published sequence datasets [5, 9]. *Diaporthe vaccinii* (CBS 160.32) of *Diaporthaceae* was used as the outgroup. All the sequences were initially aligned using CLUSTAL-X2 [25] and edited manually in MEGA-X [26]. Some characters were trimmed from both ends of the alignments. Randomized Accelerated Maximum Likelihood (RAxML) v. 8.0 [27] was used for Maximum Likelihood (ML) analysis [28] and MrBayes v. 3.2.7 for Bayesian Inference (BI) analysis [29].

ML analysis was performed using a general time reversible substitution (GTR) model with gamma-distributed rate of heterogeneity and proportion of invariant sites [30]. The model was selected with ModelTest-NG v. 0.1.7 [31] based on the Akaike Information Criterion [32]. Branch support was estimated with bootstrapping of 1000 replicates [33]. Bayesian probabilities (BP) values were defined by Markov Chain Monte Carlo (MCMC) sampling with the GTR model. Six simultaneous Markov Chains were run for 100,000 generations. The first 500 trees were discarded, and the remaining trees were used to calculate BP in the majority rule consensus tree. Phylograms were visualized using FigTree v. 1.4.4 [34]. The newly generated sequences were deposited in GenBank (Table 1). The alignments used in the analyses were submitted into TreeBase (www.treebase.org; ID: 29116).

**Table 1:** Strains used in the phylogenetic analysis with their culture accession and GenBank numbers. Strains from this study are in bold and ex-types are marked with*. NA: not available.

Species	Strain	Host	Origin	GenBank accession numbers			
				ITS	LSU	*act*	*tef1-α*
*Cytospora ailanthicola*	CFCC 89970*	*Ailanthus altissima*	China	MH933618	MH933653	MH933526	MH933494
*C*. *ampulliformis*	MFLUCC 16–0583*	*Sorbus intermedia*	Russia	KY417726	KY417760	KY417692	NA
	MFLUCC 16–0629	*Acer platanoides*	Russia	KY417727	KY417761	KY417693	NA
*C*. *amygdali*	LH357*	*Prunus dulcis*	USA	MG971853	NA	MG972002	MG971659
*C*. *atrocirrhata*	CFCC 89615	*Juglans regia*	China	KR045618	KR045700	KF498673	KP310858
	CFCC 89616	*Juglans regia*	China	KR045619	KR045701	KF498674	KP310859
*C*. *beilinensis*	CFCC 50493*	*Pinus armandii*	China	MH933619	MH933654	MH933527	MH933495
	CFCC 50494	*Pinus armandii*	China	MH933620	MH933655	MH933528	MH933496
*C*. *berberidis*	CFCC 89927*	*Berberis dasystachya*	China	KR045620	KR045702	KU710990	KU710913
	CFCC 89933	*Berberis dasystachya*	China	KR045621	KR045703	KU710991	KU710914
*C*. *bungeanae*	CFCC 50495*	*Pinus bungeana*	China	MH933621	MH933656	MH933529	MH933497
	CFCC 50496	*Pinus bungeana*	China	MH933622	MH933657	MH933530	MH933498
*C*. *californica*	9C-24*	*Juglans regia*	USA	MG971935	NA	MG972083	MG971645
*C*. *carbonacea*	CFCC 89947	*Ulmus pumila*	China	KR045622	KP310812	KP310842	KP310855
*C*. *carpobroti*	CMW 48981*	*Carpobrotus edulis*	S. Africa	MH382812	MH411216	NA	MH411212
*C*. *celtidicola*	CFCC 50497*	*Celtis sinensis*	China	MH933623	MH933658	MH933531	MH933499
	CFCC 50498	*Celtis sinensis*	China	MH933624	MH933659	MH933532	MH933500
*C*. *ceratosperma*	CFCC 89624	*Juglans regia*	China	KR045645	KR045724	NA	KP310860
	CFCC 89625	*Juglans regia*	China	KR045646	KR045725	NA	KP31086
*C*. *ceratospermopsis*	CFCC 89626*	*Juglans regia*	China	KR045647	KR045726	KU711011	KU710934
	CFCC 89627	*Juglans regia*	China	KR045648	KR045727	KU711012	KU710935
*C*. *chrysosperma*	CFCC 89629	*Salix psammophila*	China	KF765673	KF765689	NA	NA
	CFCC 89981	*Populus alba*	China	MH933625	MH933660	MH933533	MH933501
*C*. *coryli*	CFCC 53162*	*Corylus mandshurica*	China	MN854450	MN854661	NA	MN850758
*C*. *davidiana*	YW2014*	*Populus davidiana*	China	KM034870	NA	NA	NA
	CXY 1374	*Populus davidiana*	China	KM034869	NA	NA	NA
*C*. *elaeagni*	CFCC 89632	*Elaeagnus angustifolia*	China	KR045626	KR045706	KU710995	KU710918
	CFCC 89633	*Elaeagnus angustifolia*	China	KF765677	KF765693	KU710996	KU710919
*C*. *elaeagnicola*	CFCC 52883	*Elaeagnus angustifolia*	China	MK732342	NA	MK732345	NA
	CFCC 52884	*Elaeagnus angustifolia*	China	MK732343	NA	MK732346	NA
*C*. *erumpens*	CFCC 50022	*Prunus padus*	China	MH933627	MH933661	MH933534	MH933502
	MFLUCC 16–0580*	*Salix x fragilis*	Russia	KY417733	KY417767	KY417699	NA
*C*. *eucalypti*	KARE1585	*Eucalyptus globulus*	USA	MG971907	NA	MG972056	MG971617
*C*. *euonymicola*	CFCC 50499*	*Euonymus kiautschovicus*	China	MH933628	MH933662	MH933535	MH933503
	CFCC 50500	*E*. *kiautschovicus*	China	MH933629	MH933663	MH933536	MH933504
*C*. *euonymina*	CFCC 89993*	*E*.*kiautschovicus*	China	MH933630	MH933664	MH933537	MH933505
*C*. *fraxinigena*	MFLU 17–0880	*Fraxinus ornus*	Italy	MF190134	MF190079	NA	NA
*C*. *fugax*	CXY 1381	*Populus ussuriensis*	China	KM034853	NA	NA	NA
*C*. *gigalocus*	CFCC 89620*	*Juglans regia*	China	KR045628	KR045708	KU710997	KU710920
	CFCC 89621	*Juglans regia*	China	KR045629	KR045709	KU710998	KU710921
*C*. *gigaspora*	CFCC 50014	*Juniperus procumbens*	China	KR045630	KR045710	KU710999.	KU710922
	CFCC 89634*	*Salix psammophila*	China	KF765671	KF765687	KU711000	KU710923
*C*. *granati*	6F-45*	*Punica granatum*	USA	MG971799	NA	MG971949	MG971514
*C*. *hippophaes*	CFCC 89639	*Hippophae rhamnoides*	China	KR045632	KR045712	KU711001	KU710924
	CFCC 89640	*Hippophae rhamnoides*	China	KF765682	KF765698	KF765730	KP310865
*C*. *japonica*	CFCC 89956	*Prunus cerasifera*	China	KR045624	KR045704	KU710993	KU710916
*C*. *joaquinensis*	KARE975*	*Populus deltoides*	USA	MG971895	NA	MG972044	MG971605
*C*. *junipericola*	BBH 42444	*Juniperus communis*	Italy	MF190126	MF190071	NA	MF377579
	MFLU 17–0882*	*Juniperus communis*	Italy	MF190125	MF190072	NA	MF377580
*C*. *juniperina*	CFCC 50502	*Juniperus przewalskii*	China	MH933633	MH933667	MH933540	MH933508
	CFCC 50503	*Juniperus przewalskii*	China	MH933634	MH933668	MH933541	MH933509
*C*. *leucosperma*	CFCC 89622	*Pyrus bretschneideri*	China	KR045616	KR045698	KU710988	KU710911
	CFCC 89894	*Pyrus bretschneideri*	China	KR045617	KR045699	KU710989	KU710912
*C*. *leucostoma*	HigginsLake4	*Alnus incana*	USA	JX475137	NA	NA	JX438600
*C*. *longiostiolata*	MFLUCC 16–0628*	*Salix fragilis*	Russia	KY417734	KY417768	KY417700	NA
*C*. *longispora*	10F-57*	*Prunus domestica*	USA	MG971905	NA	MG972054	MG971615
*C*. *mali*	CFCC 50028	*Malus pumila*	China	MH933641	MH933675	MH933548	MH933513
	CFCC 50030	*Malus pumila*	China	MH933643	MH933677	MH933550	MH933524
*C*. *melnikii*	CFCC 89984	*Rhus typhina*	China	MH933644	MH933678	MH933551	NA
	MFLUCC 15–0851*	*Malus domestica*	Russia	KY417735	KY417769	KY417701	NA
*C*. *nivea*	MFLUCC 15–0860	*Salix acutifolia*	Russia	KY417737	KY417771	KY417703	NA
*C*. *oleicola*	KARE1021*	*Olea europaea*	USA	MG971944	NA	MG972098	MG971660
*C*. *palm*	CXY 1276	*Cotinus coggygria*	China	JN402990	NA	NA	KJ781296
*C*. *parakantschavelii*	MFLUCC 15–0857*	*Populus sibirica*	Russia	KY417738	KY417772	KY417704	NA
	MFLUCC 16–0575	*Pyrus pyraster*	Russia	KY417739	KY417773	KY417705	NA
*C*. *parapistaciae*	KARE270*	*Pistacia vera*	USA	MG971804	NA	MG971954	MG971519
***C*. *paraplurivora***	**FDS-439**	***Prunus armeniaca***	**Canada**	**OL640182**	**OL640184**	**OL631586**	**OL631589**
	FDS-564*	***Prunus persica* var. *nucipersica***	**Canada**	**OL640183**	**OL640185**	**OL631587**	**OL631590**
	**FDS-623**	***Prunus persica* var. *persica***	**Canada**	**OL640181**	**OL640123**	**OL631588**	**OL631591**
*C*. *parasitica*	MFLUCC 15–0507*	*Malus domestica*	Russia	KY417740	KY417774	KY417706	NA
	XJAU 2542–1	*Malus sp*.	China	MH798884	MH798897	NA	MH813452
*C*. *paratranslucens*	MFLUCC 15–0506*	*Populus alba var*. *bolleana*	Russia	KY417741	KY417775	KY417707	NA
	MFLUCC 16–0627	*Populus alba*	Russia	KY417742	KY417776	KY417708	NA
*C*. *pistaciae*	KARE443*	*Pistacia vera*	USA	MG971802	NA	MG971952	MG971517
*C*. *platanicola*	MFLU 17–0327*	*Platanus hybrida*	Italy	MH253451	MH253452	MH253449	NA
*C*. *platyclade*	CFCC 50504*	*Platycladus orientalis*	China	MH933645	MH933679	MH933552	MH933516
	CFCC 50505	*Platycladus orientalis*	China	MH933646	MH933680	MH933553	MH933517
*C*. *platycladicola*	CFCC 50039	*Platycladus orientalis*	China	KR045642	KR045721	KU711008	KU710931
*C*. *plurivora*	KARE1452*	*Olea europaea*	USA	MG971861	NA	MG972010	MG971572
	5L-29	*Prunus persica*	USA	MG971856	NA	MG972005	MG971567
*C*. *populicola*	KARE973*	*Populus deltoides*	USA	MG971891	NA	MG972040	MG971601
*C*. *populina*	CFCC 89644	*Salix psammophila*	China	KF765686	KF765702	KU711007	KU710930
*C*. *populinopsis*	CFCC 50032*	*Sorbus aucuparia*	China	MH933648	MH933683	MH933556	MH933520
*C*. *pruinopsis*	CFCC 50034*	*Ulmus pumila*	China	KP281259	KP310806	KP310836	KP310849
	CFCC 50035	*Ulmus pumila*	China	KP281260	KP310807	KP310837	KP310850
*C*. *pruinose*	CFCC 50036	*Syringa oblata*	China	KP310800	KP310802	KP310832	KP310845
*C*. *prunicola*	MFLU 17–0995*	*Prunus sp*.	Italy	MG742350	MG742351	MG742353	NA
*C*. *quercicola*	MFLU 17–0881	*Quercus sp*.	Italy	MF190128	MF190074	NA	NA
	MFLUCC 14–0868*	*Quercus sp*.	Italy	MF190129	MF190073	NA	NA
*C*. *ribis*	CFCC 50026	*Ulmus pumila*	China	KP281267	KP310813	KP310843	KP310856
	CFCC 50027	*Ulmus pumila*	China	KP281268	KP310814	KP310844	KP310857
*C*. *rosae*	MFLUCC 14–0845*	*Rosa canina*	Italy	MF190131	MF190075	NA	NA
*C*. *rostrata*	CFCC 89909*	*Salix cupularis*	China	KR045643	KR045722	KU711009	KU710932
*C*. *rusanovii*	MFLUCC 15–0854*	*Salix babylonica*	Russia	KY417744	KY417778	KY417710	NA
*C*. *salicacearum*	MFLUCC 15–0861	*Salix x fragilis*	Russia	KY417745	KY417779	KY417711	NA
	MFLUCC 15–0509*	*Salix alba*	Russia	KY417746	KY417780	KY417712	NA
*C*. *salicicola*	MFLUCC 15–0866	*Salix alba*	Russia	KY417749	KY417783	KY417715	NA
	MFLUCC 14–1052*	*Salix alba*	Russia	KU982636	KU982635	KU982637	NA
*C*. *salicina*	MFLUCC 15–0862*	*Salix alba*	Russia	KY417750	KY417784	KY417716	NA
	MFLUCC 16–0637	*Salix x fragilis*	Russia	KY417751	KY417785	KY417717	NA
*C*. *schulzeri*	CFCC 50040	*Malus domestica*	China	KR045649	KR045728	KU711013	KU710936
	CFCC 50042	*Malus asiatica*	China	KR045650	KR045729	KU711014	KU710937
*C*. *sibiraeae*	CFCC 50045*	*Sibiraea angustata*	China	KR045651	KR045730	KU711015	KU710938
	CFCC 50046	*Sibiraea angustata*	China	KR045652	KR045731	KU711015	KU710939
*C*. *sophorae*	CFCC 50048	*Magnolia grandiflora*	China	MH820401	MH820394	MH820409	MH820405
*C*. *sophoricola*	CFCC 89596	*Styphnolobium japonicum var*. *pendula*	China	KR045656	KR045735	KU711020	KU710943
	CFCC 89595*	*Styphnolobium japonicum var*. *pendula*	China	KR045655	KR045734	KU711019	KU710942
*C*. *sophoriopsis*	CFCC 89600*	*Styphnolobium japonicum*	China	KR045623	KP310804	KU710992	KU710915
*C*. *sorbicola*	MFLUCC 16–0584*	*Acer pseudoplatanus*	Russia	KY417755	KY417789	KY417721	NA
	MFLUCC 16–0633	*Cotoneaster melanocarpus*	Russia	KY417758	KY417792	KY417724	NA
*C*. *spiraeae*	CFCC 50049*	*Spiraea salicifolia*	China	MG707859	MG707643	MG708196	NA
	CFCC 50050	*Spiraea salicifolia*	China	MG707860	MG707644	MG708197	NA
*C*. *spiraeicola*	CFCC 53138*	*Spiraea salicifolia*	China	MN854448	MN854659	NA	MN850756
	CFCC 53139	*Tilia nobilis*	China	MN854449	MN854660	NA	MN850757
*C*. *tamaricicola*	CFCC 50507	*Rosa multifolora*	China	MH933651	MH933686	MH933559	MH933525
	CFCC 50508*	*Tamarix chinensis*	China	MH933652	MH933687	MH933560	MH933523
*C*. *tanaitica*	MFLUCC 14–1057*	*Betula pubescens*	Russia	KT459411	KT459412	KT459413	NA
*C*. *translucens*	CXY 1351	*Populus davidiana*	China	KM034874	NA	NA	NA
*C*. *ulmi*	MFLUCC 15–0863*	*Ulmus minor*	Russia	KY417759	NA	NA	NA
*Diaporthe vaccinii*	CBS 160.32	*Vaccinium macrocarpon*	USA	KC343228	NA	JQ807297	KC343954

### DNA isolation, genome sequencing and characterization

Highly purified genomic DNA from the isolate FDS-564 was extracted from fungal mycelium grown in a liquid Potato Dextrose Broth for 5 days at room temperature, filtered and freeze-dried before following the modified DNA extraction protocol as previously described (Norgen Biotech Comp., Thorold, Canada). The library was prepared using a SMRT bell Express Template Prep Kit (PacBio, Menlo Park, USA). The library pool was sequenced on one SMRT cell using the PacBio Sequel II platform in the SickKids sequencing facility (Toronto, ON, Canada). The reads were assembled into contigs with the Canu assembler v. 2.1.1 [35]. The quality of genome assemblies was accessed using QUAST v. 5.0.2 [36]. The completeness of assembly was estimated with BUSCO v.4.0.5 employing the dataset Ascomycota_odb10 [37]. Repeat sequences were identified with RepeatMasker v. 4.0.9 [38] using the library of repeats for fungi obtained from Repbase [39]. Gene prediction was executed using Augustus v. 3.4.0 [40]. Augustus was trained with the gene structures from the representative genome of *C*. *mali* 03–8 (GCA_000818155) obtained using the MAKER2 pipeline v. 2.31.11 [41].

### Phylogenomic analysis and genome alignment

The proteomes of 17 species of ascomycetes, including five species in the *Cytosporaceae*, were retrieved from the Mycocosm portal [42] (accessed May 2022) and the NCBI Genome database (accessed Dec 2021). Orthofinder v. 2.5.4 [43] was used to identify the single-copy orthogroups (SCO) in the species included in the analysis. Multiple sequence alignments were performed using MAFFT v. 7.489 [44]. The ML tree was produced with FastTree v. 2.1.10 [45]. All runs were performed using the default parameters. *Mollisia scopiformis* CBS 120377 (Leotiomycetes) was used as an outgroup (Table 2). The tree was visualized in FigTree v. 1.4.4 [34]. Genome alignment was performed with minimap2 v. 2.23 [46] and displayed as a dot-plot graph using D-Genies [47].

**Table 2:** Genomes used in phylogenomic analyses. Strains from this study are in bold. NA: not available.

Species name	Strain	GenBank assembly accession	Host	Country	Reference
*Botryosphaeria dothidea*	sdau11-99	GCA_011503125	*Malus* sp.	China	[48]
*Colletotrichum higginsianum*	IMI 349063	GCA_001672515	*Brassica rapa* subsp. *chinensis*	Trinidad and Tobago	[49]
*Cytospora chrysosperma*	CFL2056	NA	NA	Canada	PI permission
*Cytospora leucostoma*	SXYLt	GCA_003795295	*Prunus persica*	China	[50]
*Cytospora* (*Valsa*) *mali*	03–8	GCA_000818155	*Malus* sp.	China	[51]
*Cytospora* (*Valsa*) *mali* var. *pyri*	SXYL134	GCA_000813385	*Malus* sp.	China	[51]
***Cytospora paraplurivora***	**FDS-564**	**GCA_021272945**	***Prunus persica* var. *nucipersica***	**Canada**	**This study**
*Cytospora piceae*	CFCC52841	GCA_016508685	*Picea crassifolia*	China	PI permission
*Diaporthe ampelina*	DA912	GCA_001006365	*Vitis vinifera*	USA	[52]
*Diaporthe helianthi*	7/96	GCA_001702395	*Helianthus* sp.	France	[53]
*Macrophomina phaseolina*	MS6	GCA_000302655	*Corchorus olitorius*	Bangladesh	[54]
*Mollisia scopiformis*	CBS 120377	GCA_001500285	*Picea glauca*	Canada	[55]
*Neofusicoccum parvum*	UCRNP2	GCA_000385595	*Vitis vinifera*	USA	[56]
*Neurospora crassa*	FGSC 73	GCA_000786625	NA	USA	[57]
*Peltaster fructicola*	LNHT1506	GCA_001592805	*Malus* sp.	China	[58]
*Pyrenophora tritici-repentis*	Ptr	GCA_000149985	*Triticum* sp.	USA	[59]
*Pyricularia oryzae*	70–15	GCA_000002495	*Oryza sativa*	USA	[60]
*Zymoseptoria tritici*	IPO323	GCA_000219625	*Triticum* sp.	Netherlands	[61]

### Excised branch pathogenicity trials

Excised branches collected from non-symptomatic apricot, nectarine and peach trees were used to test the pathogenicity of *C*. *paraplurivora* isolates following the protocol by Arzanlou and Narmani [62]. Thirteen green lateral branches, with a mean diameter of 1 cm and 20 cm in length, were excised from each tree species. The leaves were removed, and the branches were surface disinfested with 70% ethanol for 10 min, rinsed 3 times with sterile water and air-dried. Ten branches were wounded and inoculated with 4 mm mycelium agar plug from a 5-day old cultures of each isolate and wrapped with Parafilm (Parafilm^®^ "M", MilliporeSigma, Canada). Three controls were inoculated with sterile PDA plugs. Excised branches were placed inside a clear plastic container with moist paper towels and incubated at room temperature in the dark. The branches were checked 12 days post-inoculation to measure the necrotic lesions. Longitudinal sections were made from the inoculation point for further lesion examination.

### Living plants pathogenicity trials

The peach seedlings (cv. Loring) were grown from seeds and placed in a biosafety plant growth chamber under a temperature of 23°C and a photoperiod of 16 h. A completely randomized design was used for the experiment. Inoculations were performed on four-month-old seedlings. A total of five seedlings were wounded and inoculated with 4 mm mycelium agar plug from a 5-day old cultures of each isolate and wrapped with Parafilm (Fig 7). Five seedlings were inoculated with sterile PDA plugs as negative control. The developing lesions were measured 7, 10, 13 and 27 days post-inoculation (dpi).

### Re-Isolation of the pathogen

*C*. *paraplurivora* was re-isolated from the excised branches and seedlings stem fragments and from fruiting structures (conidiomata). Three tissue fragments (0.5 cm long) were taken from the advanced necrosis and from the lesion edge of each branch and seedling. The tissue fragments were surface disinfested with 70% ethanol for 30 sec, followed by 1% NaClO for 20 min and three rinses in sterile distilled water. The samples were air-dried and placed on a 2% PDA supplemented with kanamycin (50 mg L^−1^). The PDA plates were incubated at 22°C for 5 days in the dark. All fungal colony-forming units were hyphal-tip transferred to individual PDA plates and incubated at 22°C for 7 days in the dark. One conidiomata was taken from each infected seedling and the pathogen was re-isolated using the protocol described by Chomnunti et al. [17]. The identification of *C*. *paraplurivora* re-isolated from lesions and fruiting structures was made on the basis of morphological features using reference cultures and PCR with the primer pair EF1-728F/ EF1-986R [22], as described above, to fulfil Koch’s postulates.

### Nomenclature

The electronic version of this article in Portable Document Format (PDF) in a work with an ISSN or ISBN will represent a published work according to the International Code of Nomenclature for algae, fungi and plants, and hence the new names contained in the electronic publication of a PLOS ONE article are effectively published under that Code from the electronic edition alone, so there is no longer any need to provide printed copies.

In addition, new names contained in this work have been submitted to Index Fungorum and MycoBank from where they will be made available to the Global Names Index. The unique Index Fungorum number can be resolved and the associated information viewed through any standard web browser by appending the Index Fungorum number contained in this publication to the prefix www.indexfungorum.org/. The online version of this work is archived and available from the following digital repositories: PubMed Central, LOCKSS.

## Results

The decline disease has affected up to 72% of the trees in the surveyed stone fruits orchards in southern Ontario. *C*. *paraplurivora* was the only *Cytospora* spp. isolated from symptomatic trees in all surveyed orchards. The incidence of *C*. *paraplurivora* on symptomatic trees was 50% in peaches, 57% in apricots and 100% in nectarines.

### Phylogenetic analysis

**Fig 2:**
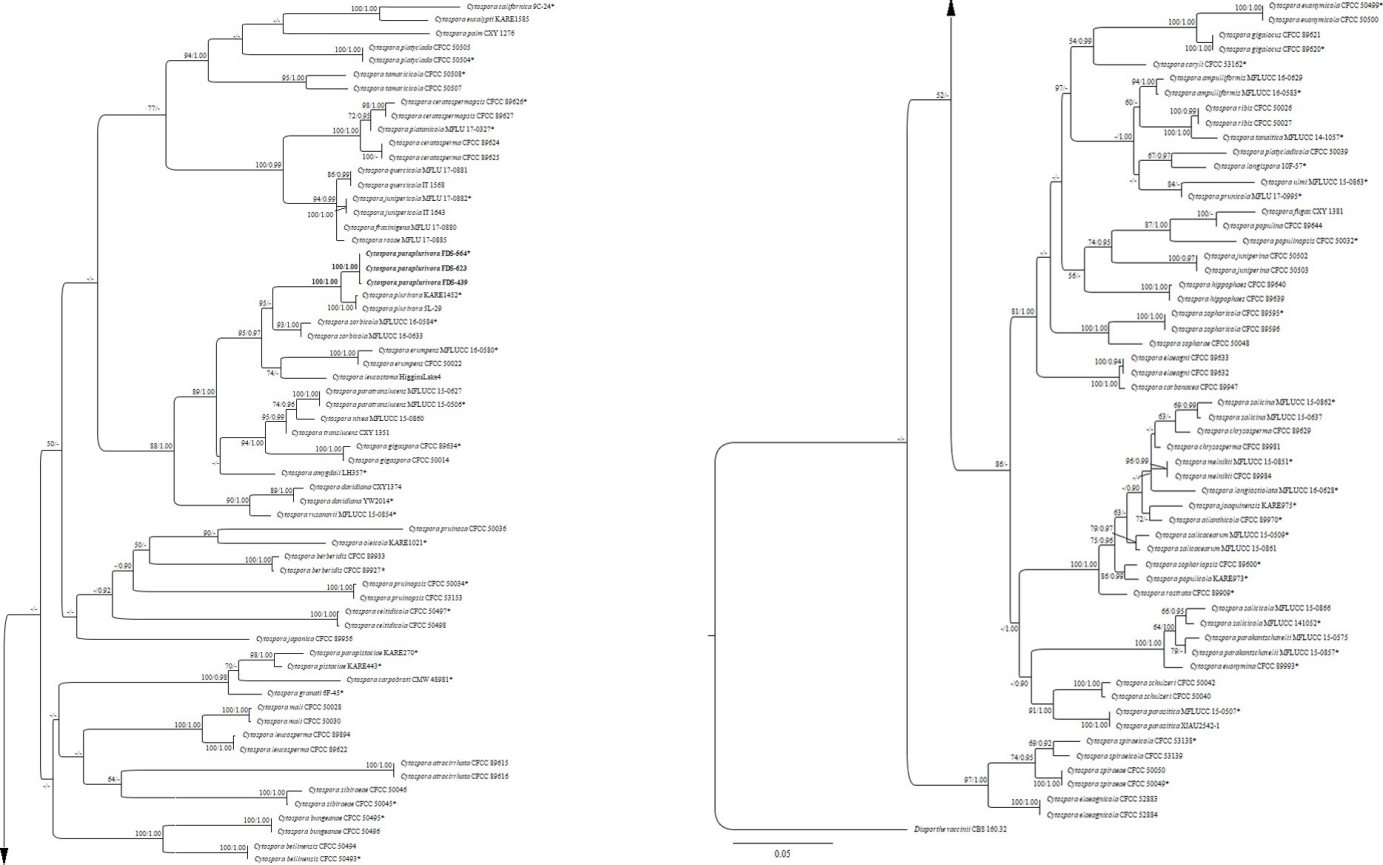
Maximum likelihood (ML) phylogeny based on a concatenated ITS, LSU, *act*, *tef*1-α sequence alignment. Bootstrap support values for ML ≥ 50% and BP ≥ 0.90 are defined as ML/BP above and below the nodes. Strains of the new species are in bold, and ex-type strains are marked with*. The tree is rooted to *Diaporthe vaccinii* (CBS 160.32).

The ML and BI analyses based on a concatenated alignment of ITS, LSU, *act* and *tef1*- α from 119 strains of *Cytosporaceae* produced phylogenetic trees with similar topology to those in recent taxonomic studies of *Cytospora* [5, 9, 12]. The best scoring RAxML tree is depicted in Fig 2. The strains of *C*. *paraplurivora* obtained in this study are clustered together and formed a distinct clade with strong bootstrap support values (100% ML, 1.00 BP). The closest species, *C*. *plurivora* is grouped with the new species in the well supported clade (100% ML, 1.00% BP). Broadly, the taxa with leucocytosporoid condiomata (*C*. *amydgali*, *C*. *davidiana*, *C*. *erumpens*, *C*. *gigaspora*, *C*. *leucostoma*, *C*. *nivea*, *C*. *paratranslucens*, *C*. *rusanovii*, *C*. *sorbicola*, *C*. *translucens*) also constitute a separate clade (88% ML, 1.00 BP).

### Taxonomy

**Fig 3:**
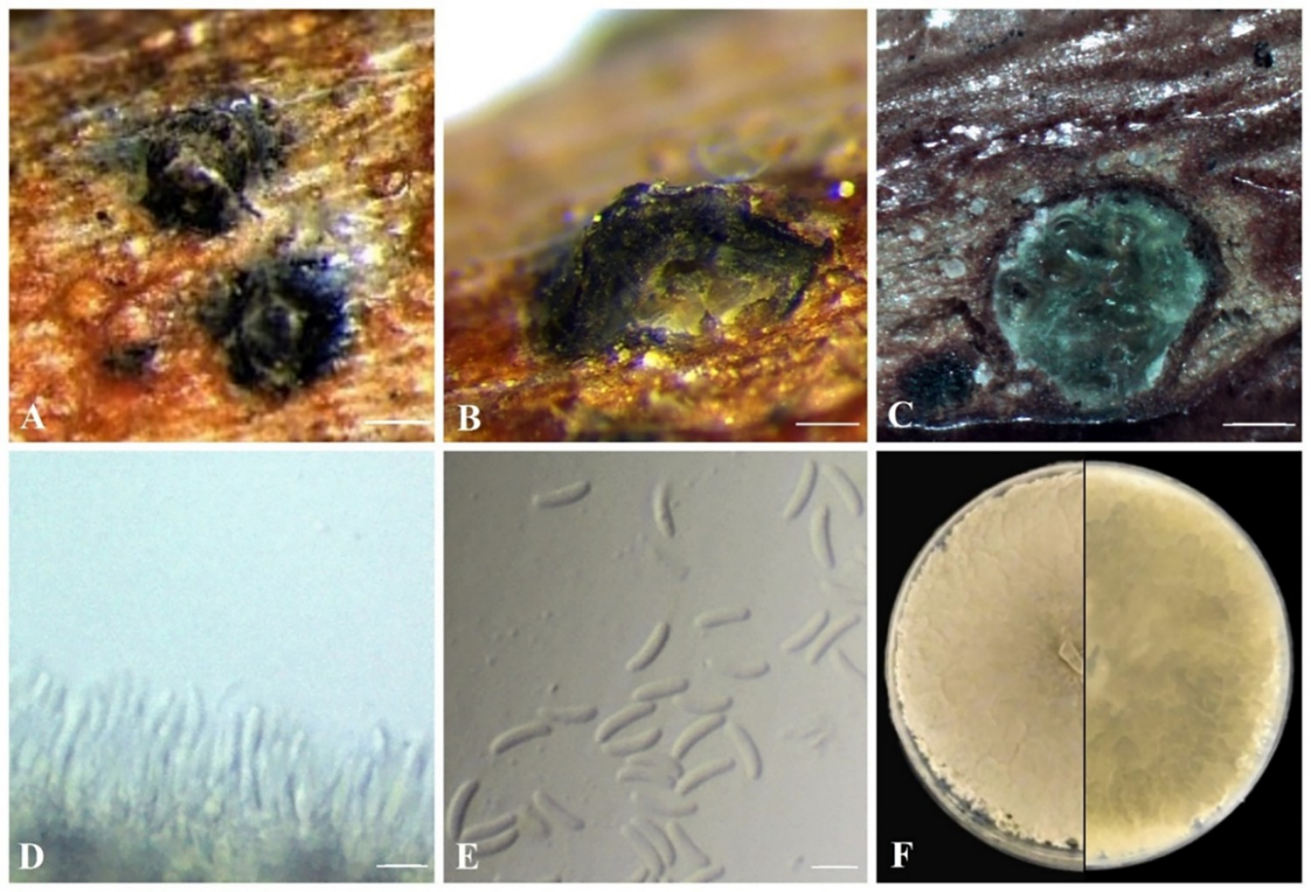
*C*. *paraplurivora* on *P*. *persica* var. *nucipersica* (FDS-564). A. Habit of conidiomata on branches. B. Longitudinal section through conidiomata. C. Transverse section of conidiomata. D. Conidiogenous cells. E. Conidia. F. Seven-day-old culture on PDA. Scale bars: A = 200 μm B-C = 100 μm, D-E = 5 μm.

***Cytospora paraplurivora*** Ilyukhin & Ellouze, sp. nov. (Fig 3).

Index Fungorum No: IF559353

Mycobank No: MB842135

Facesoffungi No: FoF19613

Etymology—the species epithet refers to Greek prefix “para-” meaning “close” and latin “plurivora” is the species name of *Cytospora plurivora*.

Holotype—DAOM 984922

Pathogenic on seedlings and branches of *Prunus persica* var. *persica*.

**Sexual morph**: not observed. **Asexual morph**: Conidiomata 440–720 × 220–310 μm diameter (n = 10), semi-immersed in host tissue, erumpent, discoid, solitary, circular to ovoid, scattered, multiloculate, with long ostiolar neck. *Ostioles* 125–160 μm diameter, at the same level as the disc or higher. *Conidiophores* unbranched, reduced to conidiogenous cells. Conidiogenous cells 7.5–10 μm (n = 20), blastic, phialidic, originate from inner layer of pycnidial wall, hyaline, smooth-walled. Conidia (4.8–)5.5–7.2(–7.4) × 1.2–1.5(–1.7) μm ($\bar{x}$ = 6.4 × 1.3 μm, n = 50), hyaline, allantoid, somewhat elongate, aseptate.

Culture characteristics: colonies on PDA are fast-growing, reaching 6.2 cm in diameter after 5 days at 25^°^C in the dark; initially white turning olivaceous after 5–7 days with thick texture at center, uneven lobate growth margin, lacking aerial mycelium; irregular, abundant pycnidia develop after 14 days. Hyphae hyaline, smooth, branched and septate.

Notes: based on morphological characteristics and phylogenetic analysis, *C*. *paraplurivora* is closest to *C*. *plurivora* isolated from different hosts in California (USA). This species is found to be associated with a canker disease of fruit (including *Prunus* spp.) and nut trees. Both species have common morphological characteristics but conidia of *C*. *paraplurivora* are notably longer than those of *C*. *plurivora* (6.4 × 1.3 μm versus 4.1 × 1.0 μm). Lack of olivaceous color of the *C*. *plurivora* cultures grown on PDA and incubated at the same conditions also differentiates these species [5].

Material examined: CANADA, southwestern Ontario, isolated from main stem of *Prunus armeniaca*, 2019, W. Ellouze, K.E. Schneider, FDS-439; southern Ontario, isolated from single conidium, pycnidia collected from branches of *Prunus persica* var. *nucipersica*, Nov. 2019, E. Ilyukhin, W. Ellouze FDS-564 (DAOM 984922 = **holotype**; DAOMC 252466 = ex-type living culture); southern Ontario, isolated from main stem of *Prunus persica* var. *persica*, Apr. 2021, W. Ellouze, K.E. Schneider, FDS-623 (DAOMC 252525 = ex-type living culture).

### Genome assembly

A total of 6 854 783 single-end long reads (NCBI SRA accession no. SRR17267775) were generated from PacBio sequencing of FDS-564 and assembled into 211 contigs (> 1000 bp in size), with an N_50_ of 658,741 bp, and a genome size of 39.7 Mb, which is within the size range of other species of *Cytosporaceae* (35.2 Mb for *C*. *mali* var. pyri SXYL134 to 41. 9 Mb for the representative genome of *C*. *mali* 03–8). Assessment of the completeness of the genome using BUSCO groups for fungi resulted in values of C:98.9% [D: 1.0%], F: 0.1%, n: 1 706 (C:complete [D:duplicated], F:fragmented, n: number of genes) which makes the genome sequence suitable for further analysis. The repeat content was 2.9% in the assembly of *C*. *paraplurivora* FDS-564. A total of 10,249 protein-coding genes were predicted, which is 9.2% fewer genes than predicted for the genome of *C*. *mali* 03–8 (11,284).

### Phylogenomic analysis

**Fig 4:**
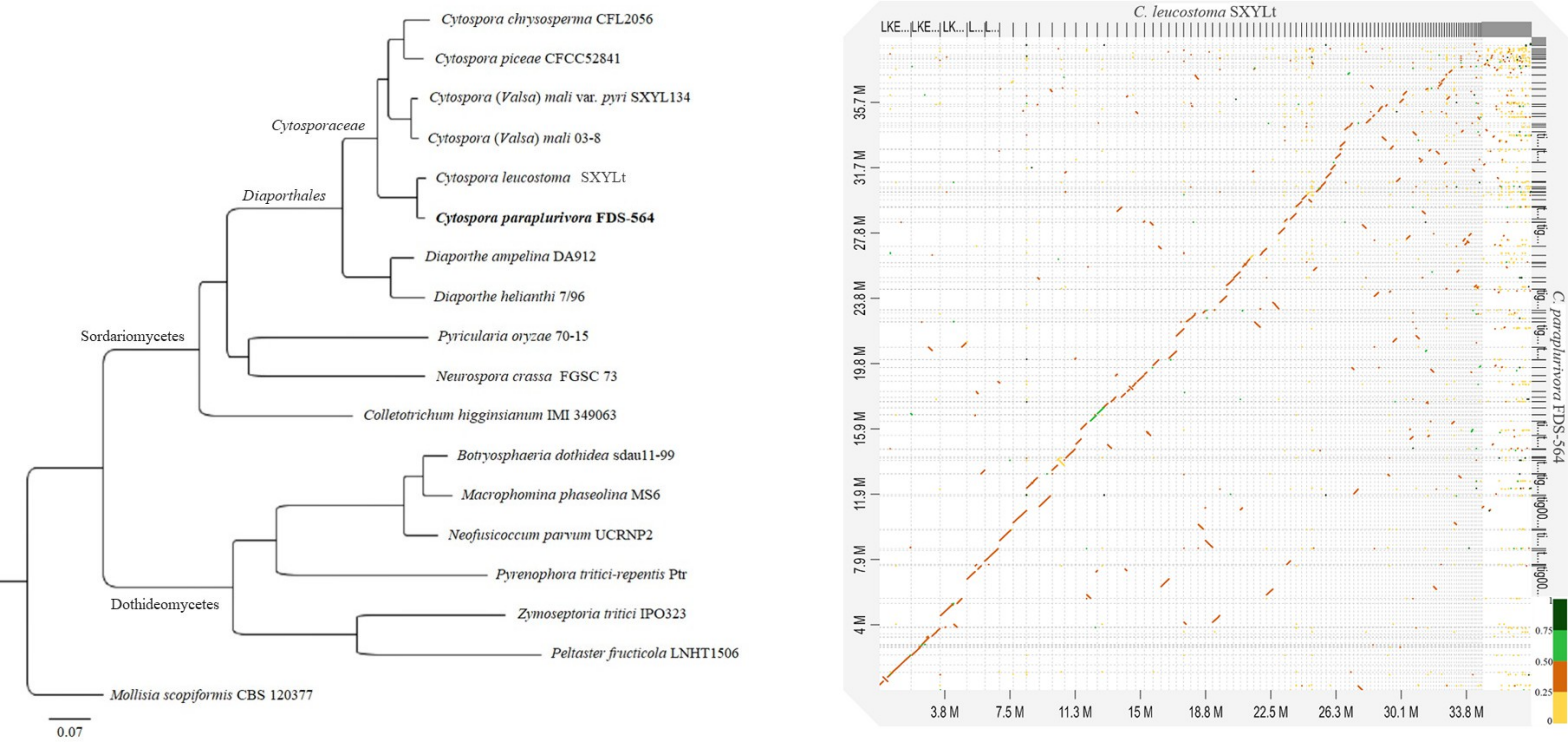
A. ML tree indicating the placement of *C*. *paraplurivora* FDS-564 among 17 species of Ascomycetes. Bootstrap support for all nodes is 100. B. Dot plot graph showing syntenic blocks between genome sequences of *C*. *leucostoma* SXYLt and *C*. *paraplurivora* FDS-564.

A total of 3,482 orthogroups present in all species included in the analysis with 2,279 SCOs. The phylogenomic tree (Fig 4A) constructed from a concatenated amino acid alignment of SCOs showed that *C*. *paraplurivora* was closest to *C*. *leucostoma* SXYLt. This analysis placed *C*. *paraplurivora* within the *Cytosporaceae* family of the order *Diaporthales* with strong bootstrap support (100% ML). However, whole genome sequence alignment between *C*. *paraplurivora* and *C*. *leucostoma* indicated rather low similarity, where 84.31% of the syntenic blocks were less than 50% similar with a number of small gaps and some repeats (Fig 4B).

### Pathogenicity tests

**Fig 5:**
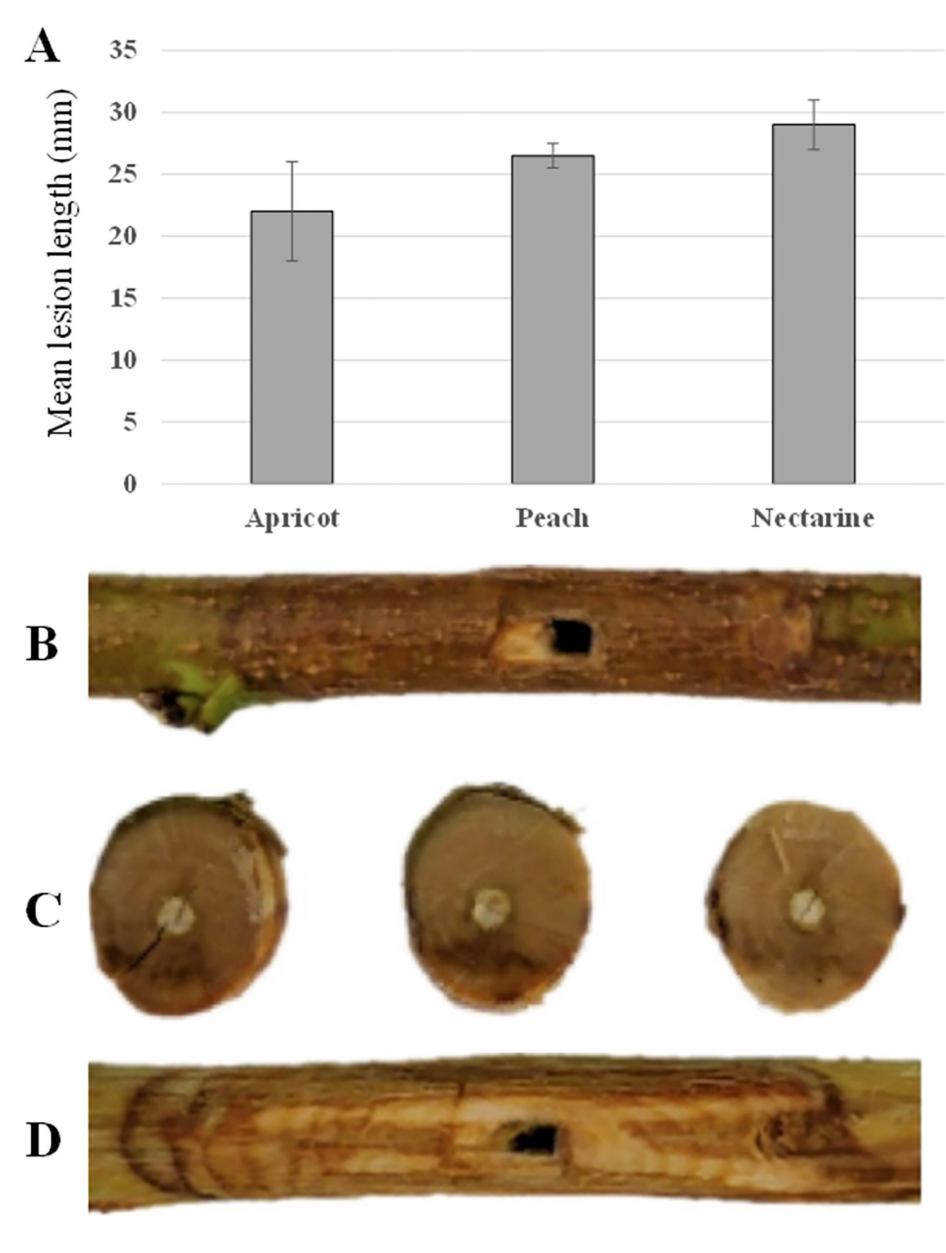
A. Mean lesion length caused by *C*. *paraplurivora* in *Prunus* spp. 12 days post-inoculation. B-D. Symptoms caused by *C*. *paraplurivora* (FDS-564) in excised branches of *P*. *persica* var. *nucipercica* 12 days post-inoculation.

**Fig 6:**
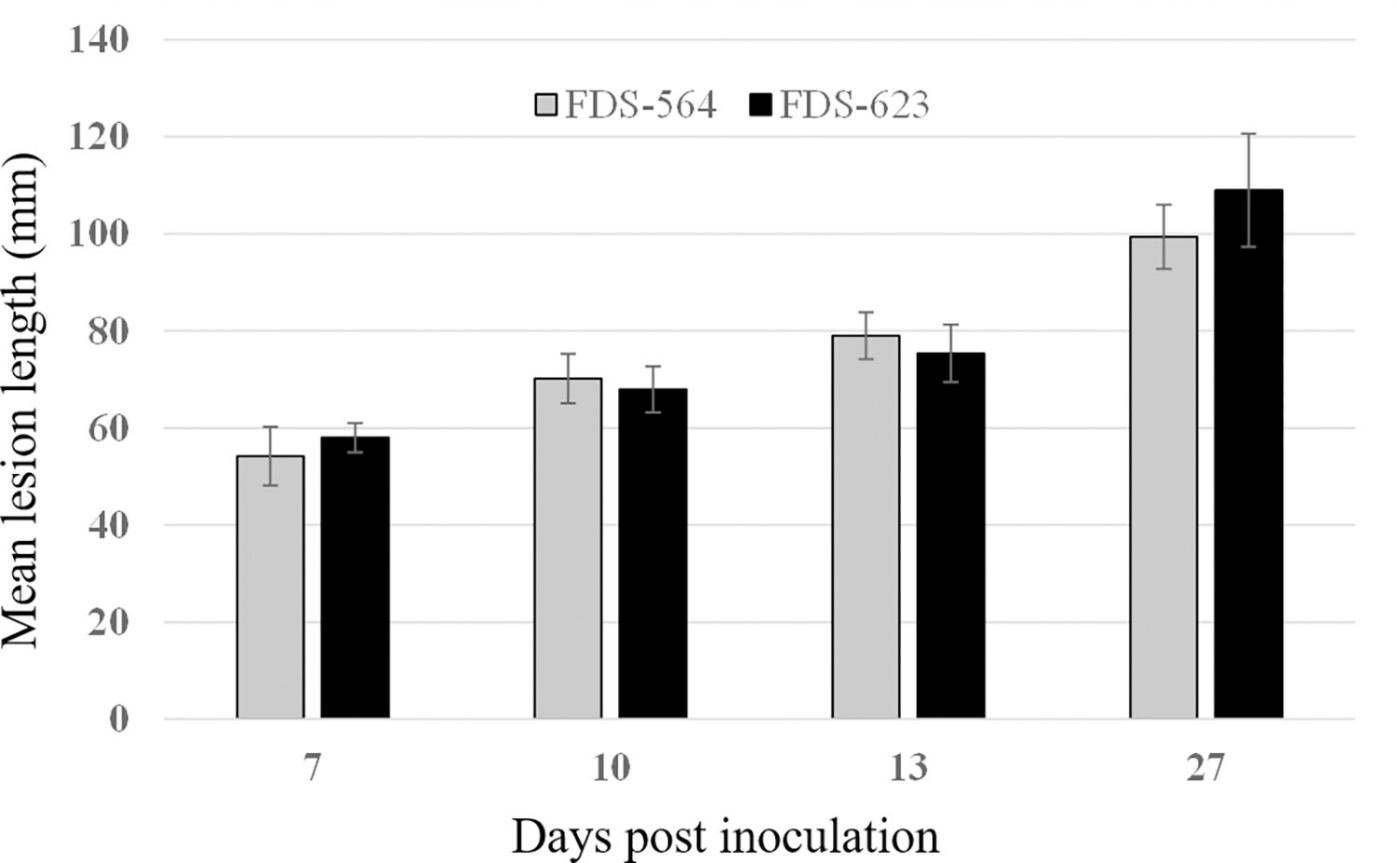
Mean lesion length caused by *C*. *paraplurivora* isolates FDS-564 and FDS-623 in *Prunus persica* var. *persica* seedlings 7, 10, 13 and 27 days post-inoculation.

**Fig 7:**
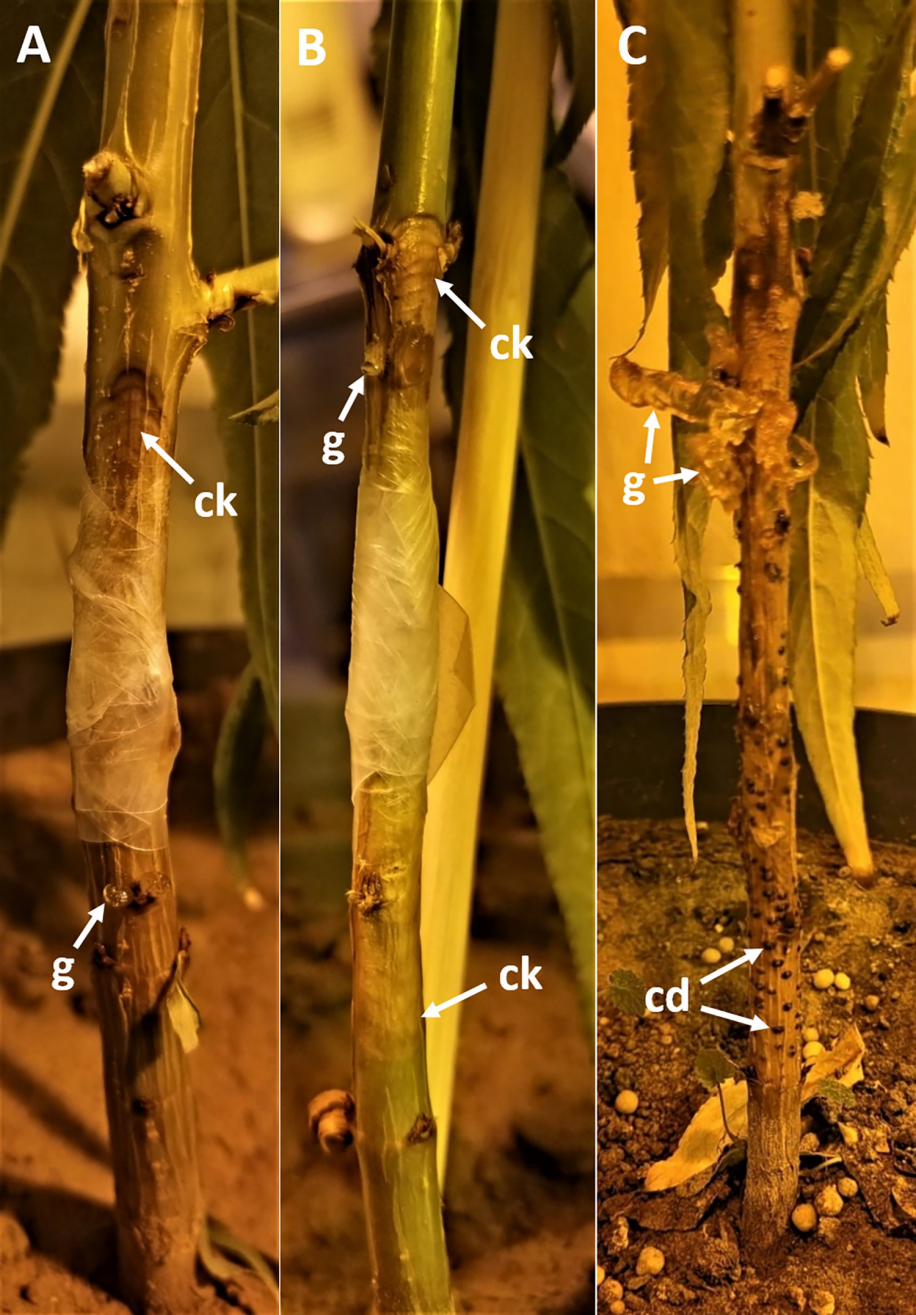
Symptoms of *Cytospora paraplurivora* infection on *Prunus persica* var. *persica* seedlings including A) 5-day old canker (ck) and gummosis (g); B) 7-day old canker; and C) 27-day old canker, conidiomata (cd) and gummosis.

Twelve days post-inoculation, the length of the necrotic lesions caused by *C*. *paraplurivora* on apricot, peach and nectarine excised branches were 22, 26 and 29 mm respectively. Lesions were significantly larger in nectarine cuttings compared to those in apricot (Fig 5A). No significant differences were observed between the mean lesion length caused by *C*. *paraplurivora* isolates FDS-564 and FDS-623 on peach seedlings 7, 10, 13 and 27 dpi (Fig 6). The canker lesions enlarged on both sides of inoculated incision. The infected peach seedlings start wilting 15 dpi and collapsed 30 dpi. The symptoms observed in the inoculated cuttings and seedlings, included flattening and discolouration of the bark, light brown wedge-shaped cankers, gummosis and abundant fruiting structures (conidiomata) development were similar to those observed on trees in the field (Figs 5B–5D and 7). *C*. *paraplurivora* was re-isolated from 90–100% of the inoculated cuttings and seedlings, fulfilling Koch’s postulates. Control cuttings and seedlings had no symptoms, and the fungus was not isolated from the wood.

## Discussion

*Cytospora* spp. are important plant pathogens with worldwide distribution causing canker and dieback diseases in woody plants. The genus has a broad host range that includes *Castanea*, *Corylus*. *Eucaliptus*, *Juglans*, *Malus*, *Olea*, *Pistacia*, *Platanus*, *Prunus*, and *Salix*. [5, 10, 12, 63, 64]. Pathogenic species of *Cytospora* were mainly isolated from wood cankers. The present study identifies *C*. *paraplurivora* sp. nov. isolated from both single conidia and cankered wood sampled from symptomatic apricot, nectarine, and peach trees in southern Ontario. Abundant conidiomata of *C*. *paraplurivora* sp. nov. were observed on trunk and branches of declining trees. *C*. *paraplurivora* sp. nov. spores dissemination mechanism might be similar to that of the sister species *C*. *plurivora* [65], other *Cytospora* spp. and fungal species causing Botryosphaeriaceae canker [66, 67]. The presence of conidiomata suggest that this species might spread across fruit tree orchards through spores dissemination by rain splash, wind and insect [65].

The symptoms caused by the new species resemble the leucostoma canker disease (perennial canker) of stone fruits, a widespread plant disease affecting crop yield in Canada and some regions of USA. *C*. *leucostoma* and *C*. *cincta* were found to be associated with this disease [68] and the species were identified solely by morphological characteristics. The studies on *Cytospora* spp. with the ITS sequence data refined the species identification of the leucocytosporoid species [69]. However, the later studies revealed that the protein-coding genes included in phylogenetic analysis allow for discrimination of closely related species of *Cytospora* [70]. In this study, using the sequence data of *act* and *tef1-α* genes suggested by Urbez-Torres et al. [39] improved the phylogenetic resolution of this species complex. This approach has also been extensively used to resolve phylogenies of other plant-associated fungal pathogens such as *Diaporthe* [71], *Valsaria* [72] and *Diplodia* [73].

Phylogenomic analysis using the genome sequence data of *C*. *paraplurivora* FDS-564 confirmed order and family level classification of the species. It is interesting to note, that the genome size of the new species is slightly larger than the average for Ascomycota (36.9Mb) [41] but significantly smaller compared to plant pathogens of *Diaporthales* such as *Daiporthe citri* 63.6Mb [74]. The high-quality genome assembly of *C*. *paraplurivora* will provide a valuable resource for the further study of host-pathogen interactions, pathogenicity-related genes, fungal biology, as well as comparative and population genomics analyses of *Cytospora* spp. and other fungal taxa.

The pathogenicity tests of *C*. *paraplurivora* on *Prunus* spp. performed in this study can be comparable to the trials of other pathogenic species of *Cytospora* on *Malus* spp. conducted *in vitro* under the same conditions with different timelines [75]. The phylogenetically close species *C*. *paratranslucens* caused necrotic lesions up to 10 cm on detached apple branches after 21 days [75]. The lesions up to 3.9 cm developed on twig segments of *Malus sieversii* inoculated with mycelium of *C*. *parasitica* after 8 days. This study showed that, highly virulent model species *C*. *mali* was able to cause the lesions up to 8.1 cm for the same time period [76]. Considering the time of post-inoculation with *C*. *paraplurivora*, it is assumed that the species can quickly colonize the plant tissue and cause the decline of infected trees. The pathogenicity tests on living peach seedlings confirmed the rapid spread of the canker lesions on the trunk of the inoculated plants, leading to the collapse of peach seedlings 30 dpi.

This paper is the first formal report for a new *Cytospora* species causing the canker of *Prunus* spp. associated with FTDS in Ontario. The findings suggest that *C*. *paraplurivora* has the potential to severely affect stone fruits production in Ontario. Accurate identification of pathogen(s) associated with stone fruit trees decline will support management of the disease. Further research needs to be conducted on emerging fungal pathogens and their associated infection mechanisms, to develop effective disease management strategies applicable to fruit tree orchards.
